# Conventional Imaging, MRI and ^18^F-FDG PET/MRI for N and M Staging in Patients with Newly Diagnosed Breast Cancer

**DOI:** 10.3390/cancers15143646

**Published:** 2023-07-17

**Authors:** Janna Morawitz, Nils-Martin Bruckmann, Kai Jannusch, Frederic Dietzel, Aleksandar Milosevic, Ann-Kathrin Bittner, Oliver Hoffmann, Svjetlana Mohrmann, Eugen Ruckhäberle, Lena Häberle, Wolfgang Peter Fendler, Ken Herrmann, Frederik Lars Giesel, Gerald Antoch, Lale Umutlu, Bernd Kowall, Andreas Stang, Julian Kirchner

**Affiliations:** 1 Department of Diagnostic and Interventional Radiology, Medical Faculty, University Dusseldorf, D-40225 Dusseldorf, Germany; nils-martin.bruckmann@med.uni-duesseldorf.de (N.-M.B.); kai.jannusch@med.uni-duesseldorf.de (K.J.); frederic.dietzel@med.uni-duesseldorf.de (F.D.); antoch@med.uni-duesseldorf.de (G.A.); julian.kirchner@med.uni-duesseldorf.de (J.K.); 2 Department of Diagnostic and Interventional Radiology and Neuroradiology, University Hospital Essen, University of Duisburg-Essen, D-45147 Essen, Germany; aleksandar.milosevic@uk-essen.de (A.M.); lale.umutlu@uk-essen.de (L.U.); 3 Department Gynecology and Obstetrics, University Hospital Essen, University of Duisburg-Essen, D-45147 Essen, Germany; ann-kathrin.bittner@uk-essen.de (A.-K.B.); oliver.hoffmann@uk-essen.de (O.H.); 4 Department of Gynecology, Medical Faculty, University Dusseldorf, D-40225 Dusseldorf, Germany; svjetlana.mohrmann@med.uni-duesseldorf.de (S.M.); eugen.ruckhaeberle@med.uni-duesseldorf.de (E.R.); 5 Institute of Pathology, Medical Faculty, University Hospital Duesseldorf, Heinrich-Heine-University, D-40204 Duesseldorf, Germany; lenajulia.haeberle@med.uni-duesseldorf.de; 6 Department of Nuclear Medicine, German Cancer Consortium (DKTK)-University Hospital Essen, University of Duisburg-Essen, D-45147 Essen, Germany; wolfgang.fendler@uk-essen.de (W.P.F.); ken.herrmann@uk-essen.de (K.H.); 7 Department of Nuclear Medicine, Medical Faculty, University Dusseldorf, D-40225 Dusseldorf, Germany; frederik.giesel@med.uni-duesseldorf.de; 8 Institute of Medical Informatics, Biometry and Epidemiology, University Hospital of Essen, D-45147 Essen, Germany; bernd.kowall@uk-essen.de (B.K.); imibe.dir@uk-essen.de (A.S.)

**Keywords:** breast cancer, metastases, staging, ^18^F-FDG PET/MRI

## Abstract

**Simple Summary:**

^18^F-FDG PET/MRI is superior in nodal staging in patients with newly diagnosed breast cancer compared to conventional imaging by sonography, CT and bone scintigraphy and compared to MRI alone. ^18^F-FDG PET/MRI correctly detects not only nodal positive status in significantly more patients, but also classifies this positive nodal status into the correct clinical lymph node stage more often than conventional imaging and than MRI alone. ^18^F-FDG PET/MRI may be a future tool as a potential alternative to invasive staging procedures for assessing the N stage. In terms of the detection of distant metastases, there is a trend towards a higher sensitivity of MRI and ^18^F-FDG PET/MRI, which, however, did not show significant differences compared with conventional staging by CT and bone scintigraphy. This demonstrates that the imaging currently recommended by multiple guidelines seems to be sufficient for the staging of distant metastases.

**Abstract:**

**Background:** This study compares the diagnostic potential of conventional staging (computed tomography (CT), axillary sonography and bone scintigraphy), whole-body magnetic resonance imaging (MRI) and whole-body ^18^F-fluorodeoxyglucose positron emission tomography (^18^F-FDG PET/)MRI for N and M staging in newly diagnosed breast cancer. **Methods:** A total of 208 patients with newly diagnosed breast cancer were prospectively included in this study and underwent contrast-enhanced thoracoabdominal CT, bone scintigraphy and axillary sonography as well as contrast-enhanced whole-body ^18^F-FDG PET/MRI. The datasets were analyzed with respect to lesion localization and characterization. Histopathology and follow-up imaging served as the reference standard. A McNemar test was used to compare the diagnostic performance of conventional staging, MRI and ^18^F-FDG PET/MRI and a Wilcoxon test was used to compare differences in true positive findings for nodal staging. **Results:** Conventional staging determined the N stage with a sensitivity of 80.9%, a specificity of 99.2%, a PPV (positive predictive value) of 98.6% and a NPV (negative predictive value) of 87.4%. The corresponding results for MRI were 79.6%, 100%, 100% and 87.0%, and were 86.5%, 94.1%, 91.7% and 90.3% for ^18^F-FDG PET/MRI. ^18^F-FDG PET/MRI was significantly more sensitive in determining malignant lymph nodes than conventional imaging and MRI (*p* < 0.0001 and *p* = 0.0005). Furthermore, ^18^F-FDG PET/MRI accurately estimated the clinical lymph node stage in significantly more cases than conventional imaging and MRI (each *p* < 0.05). Sensitivity, specificity, PPV and NPV for the M stage in conventional staging were 83.3%, 98.5%, 76.9% and 98.9%, respectively. The corresponding results for both MRI and ^18^F-FDG PET/MRI were 100.0%, 98.5%, 80.0% and 100.0%. No significant differences between the imaging modalities were seen for the staging of distant metastases. **Conclusions:** ^18^F-FDG PET/MRI detects lymph node metastases in significantly more patients and estimates clinical lymph node stage more accurately than conventional imaging and MRI. No significant differences were found between imaging modalities with respect to the detection of distant metastases.

## 1. Introduction

In 2020, more than 2.3 million people were diagnosed with breast cancer, making it the world’s most prevalent cancer, accounting for 12% of all new annual cancer cases worldwide [1,2]. Besides tumor biology, tumor stage is the most important predictive factor concerning the prognosis of breast cancer patients. While increasing the N stage not only worsens the prognosis, but also plays a role in therapeutic options such as extension of axillary surgery or extension of the radiation field in a curative therapy concept, the change from an M0 to an M1 stage usually means a change from a curative to a palliative therapy concept. The five-year survival rate of a patient with disease limited to the breast is nearly 99%, whereas the five-year survival rate worsens dramatically to 27% with the presence of distant metastases [3]. Therefore, accurate staging with detection of all affected sites of disease is crucial not only for prognosis but also for the therapeutic concept [4,5]. Whereas a few years ago axillary dissection was still regularly performed—mainly due to staging purposes—important studies such as the ACOSOG Z0011 study have shown differently: invasive lymph node dissection may be dispensed with in patients without lymph node involvement on imaging or those with up to a maximum of two affected lymph nodes on histopathological workup [6]. Since then, in latest practice, sentinel lymph node biopsy has been the gold standard for confirming nodal status [7]. While the diagnostic accuracy for T staging by sonography, mammography and also MRI has been shown to be accurate in multiple studies [8,9,10], N and M staging remains a greater challenge. In early breast cancer patients without an increased risk for distant metastases, staging focuses on the assessment of locoregional metastatic disease. According to current ESMO guidelines, patients with an increased risk for distant metastases may undergo CT of the chest, abdominal imaging (ultrasound, CT or MRI) and bone scintigraphy [11]. However, advances in imaging, particularly in hybrid imaging, have led to other modalities such as PET/CT already being investigated with regard to the staging of breast cancer patients [12]. Here, PET/CT has been shown to be superior to conventional imaging, particularly in the assessment of extra-axillary lymph node metastases [13,14]. Because of the high soft tissue contrast, MRI is highly sensitive at assessing morphologically suspicious lymph node changes such as cortex thickening or irregular margins [15,16,17]. The high soft tissue contrast also contributes to MRI’s high sensitivity in assessing distant metastases, especially bone metastases [18]. Due to the combination of the anatomical advantages of MRI, such as increased soft tissue contrast, with functional information on glucose metabolism, PET/MRI represents a promising approach for staging breast cancer, but data comparing MRI and PET/MRI with currently recommended imaging for N and M staging are still limited. 

Therefore, the aim of this study was to compare the diagnostic performance of conventional imaging modalities (axillary sonography, CT and bone scintigraphy) recommended by current guidelines with that of MRI and ^18^F-FDG-PET/MRI for N and M staging.

## 2. Materials and Methods

### 2.1. Patient Population and Inclusion Criteria

This prospective, double-center study was approved by the local ethics committees (study number 17-7396-BO and 6040R). A written informed consent form was signed by every patient prior to study enrolment. In this study, patients with newly diagnosed and treatment-naïve breast cancer, who had an increased likelihood of developing distant metastasis, were enrolled. The following criteria for an increased risk of distant metastases had to be fulfilled:

- T2 tumor or higher T stage;
- or triple negative tumor of any size;
- or tumor with molecular high-risk features (Ki67 > 14% or G3 or her2-overexpression).

All patients were included in the study between March 2018 and September 2020. Any patient who met any of the following criteria was excluded from the study: contraindications to MRI or MRI contrast agents, currently breastfeeding, pregnancy or history of malignancies within the five years prior to enrollment.

### 2.2. MRI and PET/MRI Imaging Protocol

The PET/MRI scans were conducted in a supine position, 60 min following intravenous administration of a body-weight-adjusted dose of ^18^F-FDG (4 MBq/kg body weight). All examinations were performed on an integrated Biograph mMR (Siemens Healthineers GmbH, Erlangen, Germany). To ensure the blood glucose levels were <150 mg/dL, all patients fasted for 6 h prior to the examination.

PET images were reconstructed using the iterative ordered-subset expectation maximization (OSEM) algorithm, 3 iterations and 21 subsets, a Gaussian filter with 4 mm full width at half maximum (FWHM) and a 344 × 344 image matrix. For MR-based PET attenuation correction, a two-point (fat, water) coronal 3D-Dixon-VIBE sequence was acquired to generate a four-compartment model (background air, lungs, fat, muscle). 

MRI data were acquired simultaneously with a 16-channel radiofrequency coil for the head and neck, a 24-channel radiofrequency coil for the spine and 5- or 6-channel radiofrequency coils for the body, depending on the patient’s height. The whole-body MRI protocol comprised the following sequences:

(1) A transverse T2-w half Fourier acquisition single-shot turbo spin echo (HASTE) sequence in breath-hold technique with a slice thickness of 7 mm (TE 97 ms; TR 1500 ms; Turbo factor (TF) 194; FOV 400 mm; phase FOV 75%; acquisition matrix 320 × 240 mm; in plane resolution 1.3 × 1.3 mm; TA 0:47 min/bed position);
(2) A transversal diffusion-weighted (DWI) echo-planar imaging (EPI) sequence in free breathing with a slice thickness of 5.0 mm (TR 7400 ms; TE 72 ms; b-values: 0, 500 and 1000 s/mm^2^, matrix size 160 × 90; FOV 400 mm × 315 mm, phase FOV, 75%; GRAPPA, acceleration factor 2; in plane resolution 2.6 × 2.6 mm; TA 2:06 min/bed position);
(3) A fat-saturated post-contrast transverse 3-dimensional Volumetric Interpolated Breath-hold Examination (VIBE) sequence with a slice thickness of 3 mm (TE, 1.53 ms; TR, 3.64 ms; Flip angle 9°; FOV 400 × 280 mm; phase FOV 75%; acquisition matrix 512 × 384, in plane resolution 0.7 × 0.7 mm; TA 0:19 min/bed position)

### 2.3. Conventional Staging

CT examinations were performed on dedicated CT scanners (Siemens Flash, Siemens Somatom AS, Siemens Healthineers, Erlangen, Germany). Iodinated contrast medium was administered intravenously 70 s before the scan. CT was acquired using the manufacturer-supplied dose reduction CareKV and CareDose 4D. 

Axillary sonography was conducted by experienced gynecologists (A.K.B. and S.M.) with over 10 years of expertise in breast and axillary ultrasound. The following systems and transducers were utilized: an Acuson S2000 system (Siemens Healthcare GmbH, Erlangen, Germany), a SuperSonic Imagine Aixplorer (Toshiba Medical Systems GmbH, Neuss, Germany) and an Aplio MX SSA-780A System (Toshiba Medical Systems GmbH, Neuss, Germany), all equipped with 5 to 12 MHz linear array transducers. 

Bone scintigraphy was performed with planar whole-body scans using a dual-headed gamma camera equipped with low-energy high-resolution collimator (Symbia S, Siemens Healthineers). Three hours after intravenous injection of a body-weight-adapted amount of [^99m^Tc]-labeled polyphosphonate (PDP), anterior and posterior view scans were acquired with an acquisition time of 20 to 35 min. In all cases of uncertain radionuclide accumulations in the bone scan, additional target images or SPECT/CT images were acquired.

### 2.4. Image Analysis

In every patient and modality, the lymph node status as well as distant metastases were rated as either positive or negative. In addition, the clinical N stage (cN0-cN3c) was determined for each patient using conventional imaging, MRI and ^18^F-FDG PET/MRI. According to the 8th Edition of the UICC classification, stage cN1 corresponds to the involvement of non-fixed axillary lymph nodes in level I-II, stage cN2a to the involvement of fixed axillary lymph nodes in level I–II, stage cN2b to the isolated involvement of ipsilateral internal mammary lymph nodes, stage cN3a to the involvement of infraclavicular lymph nodes (level III), stage cN3b to the involvement of axillary lymph nodes (level I–II) plus the involvement of ipsilateral A. mammaria interna lymph nodes, and stage cN3c to the involvement of supraclavicular lymph nodes [19]. 

In nodal staging, axillary sonography and CT imaging together were considered conventional imaging. In case of discrepancies in staging results between sonography and CT, the higher N stage was considered the N stage of conventional imaging. The morphological criteria used for the detection of lymph node metastases on CT and MRI included (a) short-axis diameter larger than 10 mm, (b) irregular margin, (c) heterogeneous cortex, (d) perifocal oedema, I absence of fatty hilum, (f) asymmetry compared to the contralateral site, (g) contrast media enhancement and (h) blurred nodal border [15]. In sonography, lymph nodes were considered suspicious, mostly with an indication for biopsy, if they exhibited (a) cortex thickening greater than 3 mm, (b) lobulated cortex or (c) reduced or absent hilum [20,21]. In PET/MRI, a tracer uptake higher than the direct background was indicative of malignancy. SUVmax and SUVmean values were measured by manually placing a region of interest around the respective lesion. 

In M staging, bone scintigraphy and CT together were considered conventional imaging. Lesion characterization of distant metastases was based on assessment of the preservation of anatomic boundaries/invasion of adjacent structures and assessment of contrast enhancement, and in MRI was additionally based on all T1- and T2-weighted sequences and diffusion imaging. In PET/MRI and in the bone scan, a visually detectable focal tracer enhancement over background was assessed as a possible sign of malignancy.

The analysis of conventional, MRI and ^18^F-FDG PET/MRI images was conducted in a pseudonymized manner. Two experienced radiologists specialized in hybrid imaging (J.K. and L.U.) independently reviewed the images, while the ^18^F-FDG PET/MRI images were also analyzed pseudonymized by nuclear medicine specialists (W.P.F. and F.L.G.). The analysis was performed using an OsiriX Workstation (Pixmeo SARL, Bernex, Switzerland). To prevent recognition bias, a reading intermission of 4 weeks was implemented. In cases where there were discordant readings, a collective consensus reading was conducted to resolve discrepancies. Readers were blinded to patient identity and history.

### 2.5. Reference Standard

Histopathology was employed as the definitive reference standard for evaluating the nodal status (nodal positive vs. nodal negative) in each patient. When accessible, axillary dissection or sentinel lymph node biopsy conducted prior to systemic therapy was utilized as the reference standard. In cases where an adequate pretherapeutic sample was not available, sentinel lymph node excision or axillary dissection following neoadjuvant systemic therapy was used as a surrogate reference standard. In these cases, additional histological characteristics were assessed, with focal fibrosis or focal necrosis serving as indirect indicators of previously active lymph node metastases [22,23]. For M status, histopathology served as the reference standard. If histologic sample collection was not possible, follow-up imaging was used as a reference standard (median follow-up time 13 months, range 5–32 months). In the case of metastatic lesions on imaging, at least one of the lesions was histologically confirmed. If only one of multiple suspicious lesions was confirmed histopathologically, the other suspicious lesions were also considered malignant if they met the same morphologic and/or metabolic malignancy criteria. All M0 statuses were confirmed by clinical follow-up or imaging.

### 2.6. Statistics

For statistical analyses, IBM SPSS Statistics (Version 26, IBM Deutschland GmbH, Ehningen, Germany) was used. Demographic patient data were reported using descriptive statistics. Diagnostic performance of the different modalities for N and M staging was assessed by determining sensitivity, specificity, positive predictive value (PPV), negative predictive value (NPV) and accuracy. A McNemar test was used to compare the diagnostic performance of the different imaging modalities. A Wilcoxon test was used to compare differences in true positive findings for nodal staging.

## 3. Results

### 3.1. Patient Population and Reference Standard

**Figure 1:**
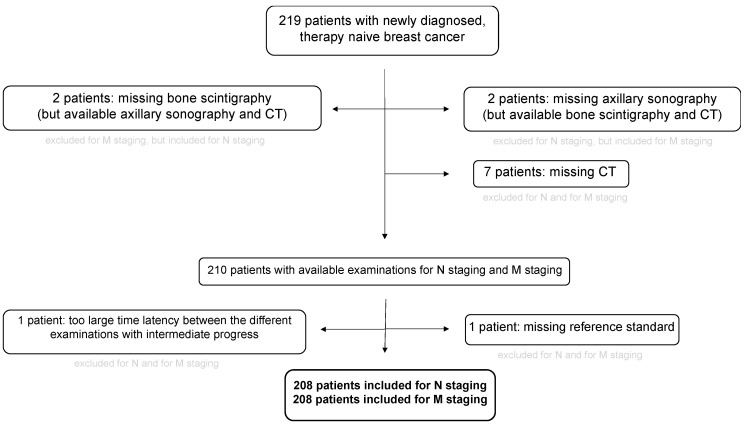
STARD-Diagram. Initial number of patients and reasons for exclusion.

A total of 208 patients were included in the study (see Figure 1). According to the reference standard, 89 patients were nodal positive (42.3%) and 119 patients were nodal negative (57.2%). Of these 89 patients, 63 patients showed a cN1 stage, 7 patients showed cN2a, 2 patients showed stage cN2b, 8 patients showed cN3a, 8 patients showed cN3c and one patient showed cN3c. In 190 patients, the nodal status was histopathologically confirmed, 100 of them pretherapeutically at the time of initial diagnosis and 90 posttherapeutically with retrospective evaluation. In 7 patients, the reference standard of nodal status was based on follow-up imaging, and in 11 patients it was decided according to expert consensus. 

A total of 196 patients did not show distant metastases (94.2%), while 12 patients showed distant metastases (5.8%). Of these 12 patients, 8 patients had bone metastases, 2 patients had liver metastases, one patient showed lymph node distant metastases and one patient suffered from pulmonary metastases. In 11 cases, distant metastases were confirmed histologically. Pulmonary metastases in one patient were confirmed by follow-up imaging. In all cases of M0 status this was confirmed by follow-up imaging. For patient demographics and tumor characteristics see Table 1. All imaging examinations were performed with a maximum time interval of 11.7 days (axillary sonography and CT 9.1 ± 7.3 days, axillary sonography and PET/MRI 11.7 ± 8.8 days, CT and PET/MRI 5.4 ± 5.6 days, bone scintigraphy and CT 4.3 ± 4.8 days, bone scintigraphy and PET/MRI 6.2 ± 6.6 days, CT and PET/MRI 6.1 ± 6.8 days).

### 3.2. N Staging

Regarding the distinction between nodal positive and nodal negative patients, conventional imaging (axillary ultrasonography and CT combined) showed a sensitivity of 80.9%, a specificity of 99.2%, a positive predictive value of 98.6%, a negative predictive value of 87.4% and an accuracy of 91.4%. The corresponding values for MRI were 78.7%, 100.0%, 100.0%, 86.2% and 90.9%, and for ^18^F-FDG PET/MRI they were 86.5%, 94.1%, 91.7%, 90.3% and 90.9% (see Table 2). These differences between conventional imaging and PET/MRI (*p* < 0.0001) and MRI and PET/MRI (*p* = 0.0005) were statistically significant. For the comparison of ^18^F-FDG PET/MRI with conventional imaging, the difference in sensitivities was Δ = 5.6% (95% KI: −5.2%; 16.4%) and the difference in specificities was ∆ = −5.0% (95% KI: −9.6%; −0.5%). For the comparison of ^18^F-FDG PET/MRI with MRI, the difference in sensitivities was Δ = 7.8% (95% KI: −3.2%; 18.9%) and the difference in specificities was Δ = 5.9% (95% KI: 1.7%; 10.1%).

**Figure 2:**
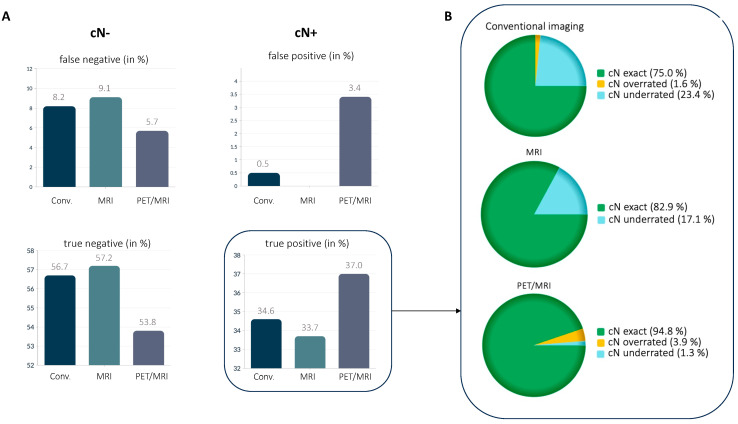
False negative, false positive, true negative and true positive detection rate of the different imaging modalities (**A**). Percentage of exact cN stage, overestimated and underestimated nodal stages among patients correctly identified as nodal positive (**B**).

**Figure 3:**
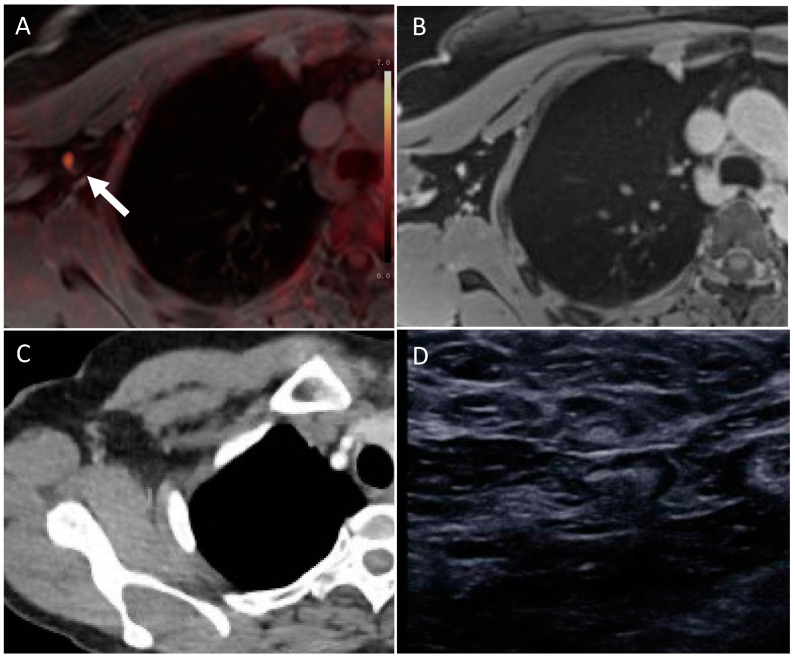
Lymph node metastases in axillary level I on the left side, correctly identified by ^18^F-FDG PET/MRI ((**A**), see white arrow), but missed by MRI (**B**), CT (**C**) and axillary sonography (**D**).

Conventional imaging detected 72/89 nodal positive patients, of whom 54 patients (54/72 = 75.0%) were classified with the correct cN stage, 17 patients (17/72 = 23.6%) with a false low cN stage and 1 patient (1/72 = 1.4%) with a false high cN stage. The corresponding values for MRI were 70/89, including 58 patients (58/70 = 82.9%) with the correct cN stage, 12 patients (12/70 = 17.1%) with a false low cN stage and no patient with a false high cN stage. ^18^F-FDG PET/MRI detected 77/89 nodal positive patients, of whom 73 patients (73/77 = 94.8%) were classified at the correct cN stage, 3 patients (3/77 = 3.9%) were falsely overclassified and 1 patient (1/77 = 1.3%) was falsely underclassified (see Figure 2). The differences between the three imaging modalities were statistically significant (each *p* < 0.05). See Figure 3 for an example of the differences in imaging modalities in the detection of lymph node metastases.

### 3.3. M Staging

Conventional imaging (CT and bone scintigraphy combined) showed a sensitivity of 83.3%, a specificity of 98.5%, a positive predictive value of 76.9%, a negative predictive value of 98.9% and an accuracy of 97.6%. The corresponding values for MRI and ^18^F-FDG PET/MRI were 100.0%, 98.5%, 80.0%, 100.0% and 98.6%, respectively (see Table 3). There were no significant differences in the differentiation between M0 vs. M1 status in ^18^F-FDG PET/MRI and MRI compared to conventional imaging (*p* = 0.705 and *p* = 0.157).

**Figure 4:**
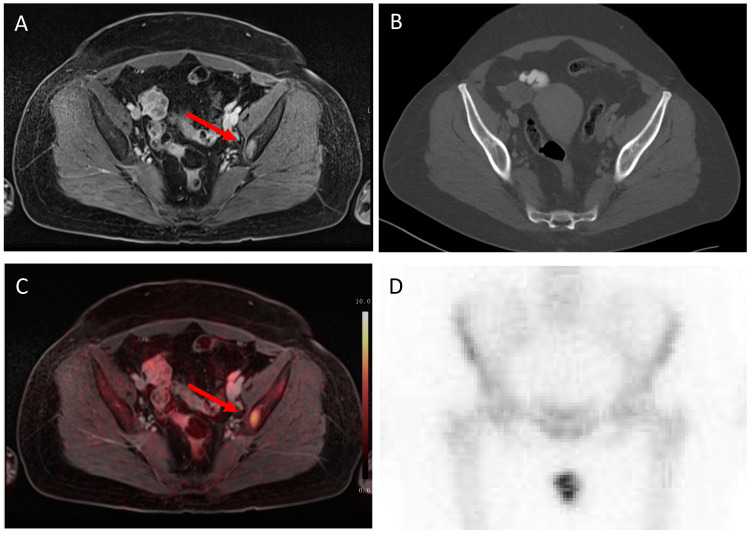
Histologically proven bone metastasis in the left Os ileum (red arrow), correctly detected by MRI (**A**) and ^18^F-FDG PET/MRI (**C**) because of suspicious contrast enhancement and focal enhanced tracer uptake (SUVmax 9.9). This bone metastasis was not detected by CT (**B**) and bone scintigraphy (**D**).

**Figure 5:**
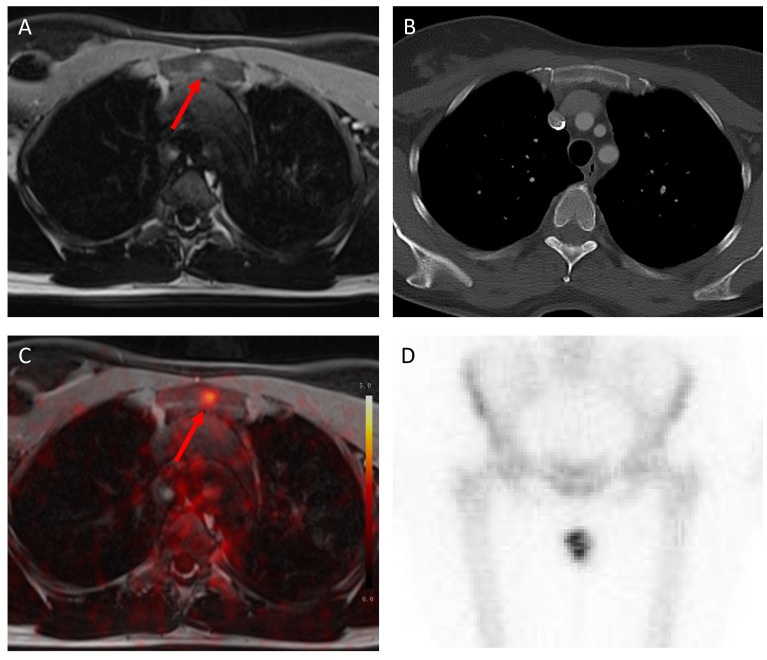
Histologically proven bone metastasis in the sternum (red arrow), correctly detected by MRI (**A**) and ^18^F-FDG PET/MRI (**C**) because suspicious contrast enhancement and focal enhanced tracer uptake (SUVmax 5.0). This bone metastasis was not detected by CT (**B**) and bone scintigraphy (**D**).

In the two cases that were detected by MRI and ^18^F-FDG PET/MRI but not by conventional imaging, the patients showed osseous metastases (see Figure 4 and Figure 5). 

## 4. Discussion

In this study, we showed that ^18^F-FDG PET/MRI detects significantly more nodal positive patients than MRI and conventional imaging and that, in the case of a positive finding, PET/MRI correctly determines the exact clinical N stage more often compared to MRI and conventional imaging. Future studies will have to address a potential impact of PET/MRI on the need for invasive procedures to stage for lymph node involvement. In addition, there was a trend toward a higher sensitivity of MRI and PET/MRI with respect to the differentiation between M0 and M1 status, but no significant difference was seen compared with conventional imaging.

The accurate detection of lymph node metastases and distant metastases is a crucial point in the primary staging of breast cancer, as this is pivotal for therapy planning. For example, if more than two affected axillary lymph nodes are detected, this means an extension of the axillary radiation field in adjuvant radiation therapy or the extension of surgical axillary treatment to an axillary dissection [24,25]. Even in the case of a negative SLNB after neoadjuvant chemotherapy, individual management of the axilla has to be discussed in a multidisciplinary team if the initial staging demonstrated axillary lymph node involvement [26]. This reflects the role of the initial (image-based) staging despite the enormous importance of SLNB. The detection of (additional) infra- and/or supraclavicular lymph nodes has no immediate surgical consequence, since these lymph nodes are usually not resected, but here too, metastatic involvement may lead to an extension of the radiation field. 

The detection of distant metastases not only has a drastic consequence in terms of prognosis, but also tailors the decision between a curative and palliative therapy concept. The results of this study are in line with previous studies, showing that MRI has a high sensitivity in the detection of distant metastases [27,28,29]. Previous studies have shown that the use of whole-body MRI in addition to CT led to changes in patient management [28]. Mainly due to the high soft tissue contrast, osseous metastases without cortical destruction are much more visible on MRI than on CT. In particular, osteolytic metastases are more difficult to detect in osteopenic bone structure, as it is increasingly found in postmenopausal women. In our study, we did not find a significant difference when differentiating between M0 and M1 status with MRI and PET/MRI. In particular, this is in line with previous studies, showing that there is no added benefit of the PET component when ^18^F-FDG PET/MRI was compared to MRI only with regard to detection of bone metastases [30]. Nevertheless, controversial results exist regarding the utility of PET/MRI in detecting distant metastases [31,32,33]. Although distant metastases often show significantly increased metabolic activity, ^18^F-FDG PET/MRI did not show superiority over MRI in our study, as all metabolically active distant metastases also showed increased contrast enhancement and/or high T2 signal on MRI and were thus also detected with MRI alone. 

The low proportion of patients with distant metastases in our study cohort may contribute to the study results indicating no significant differences when comparing the detection rates of distant metastases between (^18^F-FDG PET/)MRI and conventional staging.

Despite the superiority of CT over MRI in detecting pulmonary metastases reported in the literature, ^18^F-FDG PET/MRI was not inferior to conventional imaging here, as only one patient had pulmonary metastases that were large enough and FDG-avid such that they were also detected on ^18^F-FDG PET/MRI.

In contrast to the existing literature, in this study we not only compared individual modalities to test the superiority or inferiority of PET/MRI, but we combined different imaging modalities (sonography + CT or scintigraphy + CT as conventional imaging) to be able to transfer these study results to clinical practice, where different imaging modalities are considered together to make treatment decisions.

This study had some limitations. First, some of the histologic lymph node specimens were obtained after neoadjuvant chemotherapy and were retrospectively evaluated for prior tumor involvement. Although this method has already been described and evaluated in the literature [22,23], it carries a small residual risk of missing previously involved lymph nodes. In addition, the study design only allowed the assessment of the diagnostic performance of staging in primary breast carcinoma, which thus does not allow any statement on the diagnostic performance of the investigated modalities in re-staging and in treatment response. 

## 5. Conclusions

In conclusion, our study showed the superiority of ^18^F-FDG PET/MRI in nodal staging in patients with newly diagnosed breast cancer. ^18^F-FDG PET/MRI correctly detects not only nodal positive status in significantly more patients, but also classifies this positive nodal status into the correct clinical lymph node stage more often than conventional imaging and than MRI alone. ^18^F-FDG-PET/MRI may be a future tool as a potential alternative to invasive staging procedures for assessing the N stage.

In terms of the detection of distant metastases, there is a trend toward a higher sensitivity of MRI and PET/MRI, which, however, did not show significant differences compared with conventional staging by CT and bone scintigraphy. This demonstrates that the imaging currently recommended by multiple guidelines seems to be sufficient for the staging of distant metastases. Nevertheless, further studies with a larger number of patients with distant metastases are needed to confirm these results. 

## Figures and Tables

**Table 1:** Patient demographics and tumor characteristics.

**Total Patients**		208
**Mean age (±Standard deviation)**		54.5 ± 12.1 years
**Menopause status**		
	pre	90
	peri	15
	post	96
	unknown	7
**Ki67**		
	positive ≥ 14%	193
	negative < 14%	15
**Progesterone status**		
	positive	84
	negative	124
**Estrogen status**		
	positive	144
	negative	64
**HER2neu-expression**		
	0	78
	1+	62
	2+	29
	3+	39
**Tumor grade**		
	G1	8
	G2	105
	G3	95
**Histology**		
	NST	174
	Lobular invasive	16
	other	18

**Table 2:** Detection rate of lymph node metastases in conventional imaging, MRI and PET/MRI.

	Conventional	MRI	PET/MRI
True pos	72	70	77
True neg	118	119	112
False pos	1	0	7
False neg	17	19	12
Sensitivity	80.9 71.2–88.5	78.7 68.7–86.6	86.5 77.6–92.8
Specificity	99.2 95.4–99.9	100.0 96.9–100.0	94.1 88.3–97.6
Positive predictive value	98.6 91.1–99.8	100.0	91.7 84.2–95.8
Negative predictive value	87.4 81.9–91.4	86.2 80.8–90.3	90.3 84.6–94.1
Accuracy	91.4 86.7–94.8	90.9 86.1–94.4	90.9 86.1–94.4

**Table 3:** Diagnostic performance of conventional imaging (CT and bone scintigraphy combined), MRI and PET/MRI in differentiation between M0 and M1 status.

	Conventional	MRI	PET/MRI
True pos	10	12	12
True neg	193	193	193
False pos	3	3	3
False neg	2	0	0
Sensitivity	83.3 51.6–97.9	100 73.5–100.0	100 73.5–100.0
Specificity	98.5 95.6–99.7	98.5 95.6–99.7	98.5 96.4–99.9
Positive predictive value	76.9 51.3–91.3	80.0 60.2–96.0	80.0 60.2–96.0
Negative predictive value	98.9 96.5–99.7	100.0	100.0
Accuracy	97.6 94.5–99.2	98.6 95.8–99.7	98.6 95.8–99.7

## Data Availability

The datasets generated during and/or analyzed during the current study are available from the corresponding author on reasonable request.
